# Women’s sexual health in epidermal differentiation disorders: a call for action

**DOI:** 10.1097/JW9.0000000000000250

**Published:** 2026-02-11

**Authors:** Nessa Aghazadeh Mohandesi, Elika D. Javaheri, Amy S. Paller

**Affiliations:** a Departments of Dermatology, Clinical Genomics and Pediatrics, Mayo Clinic, Rochester, Minnesota; b Mayo Clinic Alix School of Medicine, Phoenix, Arizona; c Department of Dermatology, Northwestern University Feinberg School of Medicine, Chicago, Illinois

**Keywords:** epidermal differentiation disorders, genodermatoses, sexual health, women’s health

**What is known about this subject in regard to women and their families?**

- Women’s sexual health is an understudied topic in dermatology. Though recent studies have investigated the role of chronic dermatoses such as hidradenitis suppurativa on women’s sexual health, minimal research has characterized the effect of genodermatoses, such as epidermal differentiation disorders, on sexual wellbeing. Patients with epidermal differentiation disorders may experience vulvovaginal dryness, infections, and dyspareunia, potentially interfering with hygiene, use of menstrual products, and sexual activity.

**What is new from this article as messages for women and their families?**

- This article draws attention to the sexual health needs of women with genodermatoses. Further research is required to understand the vulvovaginal involvement of these conditions, examine patient-reported experiences, and create consensus guidelines of care.

Women’s sexual health is an essential component of overall well-being, yet it has historically been underrecognized and undertreated in both clinical practice and society. While sexual health has gained some attention in relation to chronic skin conditions, the effect of genetic skin disorders on sexual health remains largely unexplored.^1,2^ This underrepresentation not only highlights a gap in dermatologic research but also raises important questions about how sexuality, identity, and bodily autonomy are addressed in individuals with genetic skin conditions.

Epidermal differentiation disorders (EDDs) are a group of inherited disorders (formerly called ichthyosis and palmoplantar keratoderma) characterized by impaired differentiation and disrupted epidermal barrier function. EDDs may chiefly affect the integumentary system (termed “nonsyndromic EDDs”) or may affect multiple physiologic systems (termed “syndromic EDDs”) (see Supplementary Information, https://links.lww.com/IJWD/A82). However, the effect of EDDs on the vulvovaginal region has not been systematically studied. Possible vulvovaginal manifestations include dryness, fissuring, maceration, malodor, pruritus, pain, and recurrent infections.^3^ These manifestations may affect hygiene, quality of life, use of menstrual products, and physical comfort, thereby impacting sexual health.^1,2^

Individuals with EDDs may also face multiple barriers to sexual intimacy, including dyspareunia, sensitivity to lubricants, and frequent infections.^1^ Psychosocial factors, such as fear of pain, altered body image, low self-esteem, and partner misunderstanding, may further limit sexual well-being.^1^ Adolescents are particularly vulnerable as they navigate sexual identity and relationships, while coping with a visible, stigmatized condition; female adolescents may feel especially self-conscious due to heightened societal beauty standards for women.

Physician-related barriers also contribute to this gap. Dermatologists may lack training, skills, time, or confidence to discuss anogenital symptoms or sexual concerns, especially with adolescent patients. In a survey of patients with chronic skin diseases and genital involvement, 78.1% reported that clinicians did not adequately inquire about their genital symptoms or intimacy concerns.^4^ Adolescents and young adults with chronic genetic skin conditions demonstrate low readiness for transition of care, particularly in psychosocial domains, which include sexual health and intimacy.^5^ Sexual concerns are frequently deprioritized during the vulnerable transition period, and care becomes fragmented, leaving patients without consistent support during a critical developmental period for sexual health.

Drawing on previous literature, we identify 4 major barriers to achieving sexual health equity for female patients with EDDs (Fig. 1):^1^

**Fig. 1.**
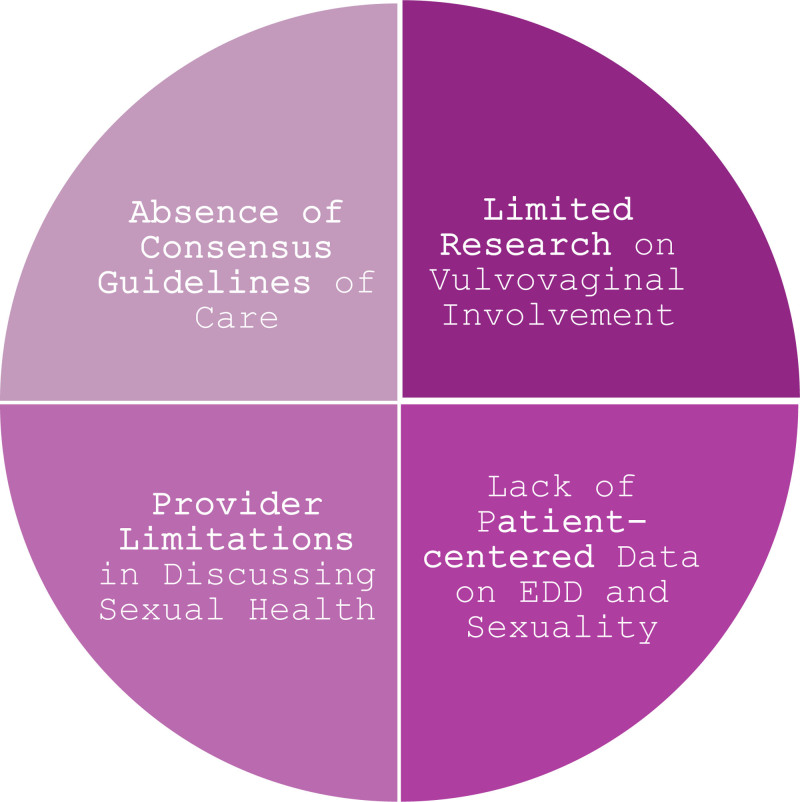
Diagram of barriers to achieving sexual health equity in patients with epidermal differentiation disorders.

1. Limited research on vulvovaginal involvement of EDDs.
2. Lack of patient-centered data on the lived experiences of individuals with EDDs and sexuality.
3. Physician limitations in discussing sexual health, especially with adolescents.
4. Absence of best practice and consensus guidelines of care.

Addressing women’s sexual health in genetic skin diseases, including EDDs, is long overdue. By engaging patients, encouraging focused research, and integrating sexual health into dermatologic care, dermatologists can begin to close this gap. Individuals with EDDs deserve care that treats more than just their skin; they deserve support for their full identity, including their right to sexual well-being.

## Conflicts of interest

The authors made the following disclosures: A.S.P.: Investigator or consultant for AbbVie, Abeona, Arcutis, BioCryst, BioMendics, Boehringer Ingelheim, Castle Creek, Chiesi, Daiichi Sankyo, Dermavant, Eli Lilly, Galderma, Incyte Corporation, Johnson & Johnson Innovative Medicine, Krystal Biotech, LEO Pharma, L’Oreal, MoonLake, Peltheos, Quoin, Regeneron, Sanofi, and UCB. None are relevant to this study.

## Funding

None.

## Study approval

N/A

## Author contributions

NAM: Conceived the paper idea, contributed to manuscript writing, and conducted the literature review. EDJ: Contributed to manuscript writing and literature review. ASP: Supervised the project and provided critical review and revisions of the manuscript.

## Supplementary data

Supplementary material associated with this article can be found at https://links.lww.com/IJWD/A82.

## Supplementary Material
