# Supplementary material for: Women’s sexual health in epidermal differentiation disorders: a call for action
Source: Int J Womens Dermatol. 2026 Feb 11;12(1):e250. doi: 10.1097/JW9.0000000000000250 (PMC12900178; doi:10.1097/JW9.0000000000000250)
Supplement: Supplementary file 1 [file jw9-12-e250-s001.pdf]

Supplemental Information

Reference 1: Paller AS, Teng J, Mazereeuw-Hautier J, et al. Syndromic epidermal differentiation disorders: a new classification toward pathogenesis-based therapy. Br J Dermatol. . 2025;193(4):592-618. doi:10.1093/bjd/ljaf123

Reference 2: Akiyama M, Choate K, Hernandez-Martin A, et al. Nonsyndromic epidermal differentiation disorders: a new classification toward pathogenesis-based therapy. Br J Dermatol. . 2025;193(4):619-641. doi:10.1093/bjd/ljaf154

Reference 3: Paller AS, Akiyama M, Hernandez-Martin A, Mazereeuw-Hautier J & Sprecher E (2025). New gene-based classification of ichthyoses and palmoplantar keratodermas: hereditary epidermal differentiation disorders.. Journal of the American Academy of Dermatology, https://dx.doi.org/10.1016/j.jaad.2025.08.086

Reference 4: Hernandez-Martin A, Paller AS, Sprecher E, Akiyama M, Mazereeuw-Hautier J. Proposing an immune-inclusive lens to the new epidermal differentiation disorders classification: reply from authors. Br J Dermatol. . 2025;193(4):800-802. doi:10.1093/bjd/ljaf188
